# “I need more knowledge”: Qualitative analysis of oncology providers’ experiences with sexual and gender minority patients

**DOI:** 10.3389/fpsyg.2022.763348

**Published:** 2022-08-15

**Authors:** Christina L. Tamargo, Edith P. Mitchell, Lynne Wagner, Melissa A. Simon, Ruth C. Carlos, Bruce J. Giantonio, Matthew B. Schabath, Gwendolyn P. Quinn

**Affiliations:** ^1^Department of Internal Medicine, Johns Hopkins Hospital, Baltimore, MD, United States; ^2^Department of Medical Oncology, Thomas Jefferson University, Philadelphia, PA, United States; ^3^Department of Social Sciences and Health Policy, Wake Forest University, Winston-Salem, NC, United States; ^4^Department of Obstetrics and Gynecology, Robert H. Lurie Comprehensive Cancer Center, Feinberg School of Medicine, Northwestern University, Chicago, IL, United States; ^5^Department of Radiology, University of Michigan, Ann Arbor, MI, United States; ^6^Department of Hematology/Oncology, Massachusetts General Hospital, Boston, MA, United States; ^7^Department of Cancer Epidemiology, H. Lee Moffitt Cancer Center & Research Institute, Tampa, FL, United States; ^8^Department of Obstetrics and Gynecology and Population Health, NYU Grossman School of Medicine, New York, NY, United States

**Keywords:** cancer, oncology, clinicians, healthcare disparities, sexual and gender minorities, LGBT

## Abstract

**Background:**

While societal acceptance for sexual and gender minority (SGM) individuals is increasing, this group continues to face barriers to quality healthcare. Little is known about clinicians’ experiences with SGM patients in the oncology setting. To address this, a mixed method survey was administered to members of the ECOG-ACRIN Cancer Research Group.

**Materials and methods:**

We report results from the open-ended portion of the survey. Four questions asked clinicians to describe experiences with SGM patients, reservations in caring for them, suggestions for improvement in SGM cancer care, and additional comments. Data were analyzed using content analysis and the constant comparison method.

**Results:**

The majority of respondents noted they had no or little familiarity with SGM patients. A minority of respondents noted experience with gay and lesbian patients, but not transgender patients; many who reported experience with transgender patients also noted difficulty navigating the correct use of pronouns. Many respondents also highlighted positive experiences with SGM patients. Suggestions for improvement in SGM cancer care included providing widespread training, attending to unique end-of-life care issues among SGM patients, and engaging in efforts to build trust.

**Conclusion:**

Clinicians have minimal experiences with SGM patients with cancer but desire training. Training the entire workforce may improve trust with, outreach efforts to, and cancer care delivery to the SGM community.

## Introduction

Sexual and gender minority (SGM) populations include, but are not limited to, those who identify as lesbian, gay, bisexual, transgender, or queer (LGBTQ), as well as asexual, intersex, and/or two-spirit; individuals with same-sex or -gender attractions or behaviors, those with differences in sexual development, and those who identify with non-binary constructs of sexual orientation, gender, or sex are also included (Sexual and Gender Minority Research Office, 2019). Roughly 4.5% of the United States population, which amounts to over 11 million people, is estimated to identify as LGBT, though this may not include other SGM populations that do not identify as cisgender LGB or transgender (Conron and Goldberg, 2020). SGM populations face a multitude of health disparities compared to cisgender heterosexual populations, stemming from issues including increased poverty (Badgett et al., 2019), denial of care due to their sexual or gender identity (Lambda Legal, 2010), fears related to discrimination (Eckstrand and Potter, 2017; McNeill et al., 2021), and inadequate training by healthcare professionals (Lambda Legal, 2010), among others.

In addition to facing barriers to quality healthcare, SGM patients have unique medical concerns in multiple areas, including oncology (Quinn et al., 2015). Many cancers disproportionately affect SGM patients, which is attributed to higher prevalence of risk factors like alcohol use and obesity, reduced cancer screening, and the aforementioned barriers to care (Institute of Medicine Committee on Lesbian G, Bisexual, and Transgender Health Issues and Research Gaps and Opportunities, 2011; Machalek et al., 2012; Agénor et al., 2014; Quinn et al., 2015; Tabaac et al., 2018; Charkhchi et al., 2019). Despite these well-described health disparities among SGM patients, there is a deficiency of research on SGM patient populations, evidence-based guidelines regarding oncologic care in SGM patients, and training on SGM-related cancer care (Quinn et al., 2015; Sutter et al., 2020).

As oncology providers play essential roles in SGM patients’ interactions with the healthcare system, examining their knowledge and attitudes regarding SGM cancer patients may shed light on the current state of the healthcare system and identify specific areas for improvement regarding SGM patient care. Prior studies by our group conducted among oncologists at National Cancer Institute (NCI)-designated cancer centers demonstrated that oncology providers are generally comfortable with sexual minority patients, but less so with specific gender minorities such as transgender patients. Additionally, these studies demonstrated that knowledge about SGM-specific oncology healthcare needs is limited, but oncologists expressed interest in receiving education and training about such issues (Shetty et al., 2016; Tamargo et al., 2017; Schabath et al., 2019; Sutter et al., 2020). Building on our prior work that focused on oncologists at NCI-Designated Cancer Centers, the current study was conducted among a more diverse population of providers that included oncologists, nurses, and physician assistants who are members of the ECOG-ACRIN Cancer Research Group (merger of Eastern Cooperative Oncology Group and American College of Radiology Imaging Network) and practice medicine at diverse academic and non-academic medical centers. The current study reports the results from the qualitative portion of the survey.

Although other studies have examined barriers to healthcare for SGM populations, including in the field of oncology, there are limitations to existing research. First, many United States studies are from the perspective of SGM individuals rather than healthcare providers, or are combined studies with limited responses from healthcare providers (Stover et al., 2014; Agénor et al., 2015; Simoni et al., 2017; Burton et al., 2020). With the exception of a recent study by Ussher et al., very few studies of healthcare providers are as large or encompass multiple types of healthcare providers (i.e., nurses, physicians, etc.; Carabez et al., 2015; Bjarnadottir et al., 2019; Burton et al., 2020; Sutter et al., 2020; Ussher et al., 2021). Finally, no studies thus far have examined qualitative comments on provider attitudes and behaviors to this extent. This study seeks to bridge that gap in research by performing an in-depth analysis of all qualitative comments from a large quantity of multiple types of oncology providers.

## Materials and methods

### Study population and survey design

We administered a web-based survey to members of the ECOG-ACRIN Cancer Research Group in late 2019. The validated survey was developed from published surveys on the knowledge, attitudes, and practice behaviors of clinicians regarding providing cancer care to SGM individuals, and has been revised and utilized by our group in other studies (Bonvicini and Perlin, 2003; Garcia, 2003; Kelley et al., 2008; Kitts, 2010; Reed et al., 2010; Abdessamad et al., 2013; Lim et al., 2013; Schabath et al., 2019). The survey included 19 demographic questions, 12 items on attitudes toward treating SGM patients, seven SGM-related knowledge questions, four practice-related questions focusing on intake forms, and four open-ended questions. The open-ended questions were, “Please describe any personal experiences treating LGBTQ patients that you consider important or informative,” “Please explain any reservations in treating the LGBTQ population,” “What suggestions do you have for improving the cancer care of the LGBTQ population?” and “Please provide any additional comments.” We report here on the results of the open-ended questions.

### Analysis

Inductive and deductive content analyses as well as the constant comparison method were used to guide analysis (Elo and Kyngäs, 2008; Constant Comparison, 2011). Two members of the team conducted the coding and analysis process. First, using the survey questions, one team member performed open coding to develop an initial codebook using the *a priori* themes from the survey questions. Next, each team member separately attempted to apply the *a priori* codes from the original list to 25 survey responses with the additional goal of identifying any new or emergent themes. Then the two coders met to compare their coding and discuss emergent themes. The code list was then revised, emergent themes were added to the list and applied again to another 25 responses, and conflicts were resolved through discussion. Once the two coders had reached an acceptable interrater reliability rate (81%; McHugh, 2012), then each coder read all 558 survey responses, and both team members independently identified themes associated with each response. Final differences in coding were resolved *via* discussion among team members until consensuses were reached. Finally, the coders reviewed all coding from each of the four questions and chose the most commonly reported and unifying themes to highlight in the manuscript.

## Results

Among the 490 healthcare providers who responded to the survey, 228 (46.5%) provided responses to one or more open-ended questions, amounting to 558 total individual responses. Among respondents who reported their demographic information, the average age was 48.3 (SD 12.1), and most identified as white (74.6%), non-Hispanic/Latino (89.0%), heterosexual (81.1%), Christian (53.9%), and female (73.2%; Table 1). Over one-third (37.3%) were registered nurses, followed by 30.3% who were licensed medical doctors specializing predominantly in hematology and/or oncology. The majority of respondents (60.1%) reported seeing zero to 25 patients per week, and the greatest proportion (46.1%) approximated that 1–5% of their patients in the last year had identified as LGBTQ.

**Table 1:** Characteristics of clinicians who responded to open-ended question(s).

Characteristic	
**Age, mean (SD)**	48.3 (12.1)
**Gender, *n* (%)**	
Female	167 (73.2)
Male	48 (21.1)
Male-to-female transgender	1 (0.4)
Prefer not to answer	11 (4.8)
Did not answer	1 (0.4)
**Sexual orientation, *n* (%)**	
Heterosexual	185 (81.1)
Bisexual	8 (3.5)
Gay	6 (2.6)
Lesbian	6 (2.6)
Other	2 (0.9)
Prefer not to answer	18 (7.9)
Did not answer	3 (1.3)
**Race, *n* (%)**	
White/Caucasian	170 (74.6)
Multiracial	14 (6.1)
Black/African–American	12 (5.3)
Asian	9 (3.9)
American Indian/Alaska Native	1 (0.4)
Hawaiian/Pacific Islander	1 (0.4)
Other/not sure	1 (0.4)
Prefer not to answer	17 (7.5)
Did not answer	3 (1.3)
**Ethnicity, *n* (%)**	
Not hispanic/Latino	203 (89.0)
Hispanic/Latino	7 (3.1)
Prefer not to answer	17 (7.5)
Did not answer	1 (0.4)
**Religious identity, *n* (%)**	
Christian	123 (53.9)
Not religious	32 (14.0)
Atheist/agnostic	27 (11.8)
Jewish	6 (2.6)
Hindu	5 (2.2)
Muslim	4 (1.8)
Buddhist	1 (0.4)
Other	10 (4.4)
Prefer not to answer	18 (7.9)
Did not answer	2 (0.9)
**Political leaning, *n* (%)**	
Liberal	62 (27.2)
Somewhat Liberal	32 (14.0)
Centrist/moderate	25 (11.0)
Very liberal	24 (10.5)
Conservative	22 (9.6)
Somewhat conservative	18 (7.9)
Very conservative	3 (1.3)
Other	5 (2.2)
Prefer not to answer	36 (15.8)
Did not answer	1 (0.4)
**Geographic region, *n* (%)**	
East North Central	50 (21.9)
Middle Atlantic	37 (16.2)
West North Central	33 (14.5)
South Atlantic	28 (12.3)
New England	24 (10.5)
Pacific	19 (8.3)
East South Central	14 (6.1)
West South Central	13 (5.7)
Mountain	8 (3.5)
Did not answer	2 (0.9)
**Practice setting** ^a^	
Main campus of AMC^b^/Medical School	109 (47.8)
Community Hospital	59 (25.9)
NCORP^c^ community site	43 (18.9)
Medical center not affiliated with medical school	21 (9.2)
Office-based	32 (14.0)
Satellite clinic of AMC^b^	12 (5.3)
NCORP^c^ minority/underserved site	9 (3.9)
VA or other government entity	1 (0.4)
Other	12 (5.3)
**Licensure** ^a^	
Registered Nurse (RN)	85 (37.3)
Doctor of Medicine (MD)	69 (30.3)
Doctor of Philosophy (PhD)	7 (3.1)
Nurse Practitioner (NP)	7 (3.1)
Doctor of Osteopathic Medicine (DO)	2 (0.9)
Physician Assistant (PA)	1 (0.4)
Other	45 (19.7)
Prefer not to answer	17 (7.5)
Did not answer	22 (9.6)

a Able to give multiple answers.

b Academic Medical Center.

c National Cancer Institute Oncology Research Program.

We identified multiple themes from the 558 responses. The major themes we highlighted were lack of experience treating SGM patients, challenges related to gender identification and pronoun use, providers’ perceptions of SGM patient attitudes, positive experiences with SGM populations, end-of-life issues related to SGM oncologic care, specific clinical care scenarios involving SGM populations, and the need for education and training (Supplementary Table 1).

### Lack of experience

Providers may feel uncomfortable when treating, or be unprepared to treat, SGM patients because they have limited experience with this patient population. Furthermore, even when they do interact with SGM patients, they may not be aware of the patients’ sexual orientations or gender identities. One provider reported this experience precisely:

“I have no experience speaking with patients of the LGBTQ community. If I did, it was not [to] my knowledge.”

Notably, many providers indicated limited exposure to some SGM patient populations, particularly transgender patients, but greater familiarity with others, such as gay and lesbian patients:

“I have had limited experience with transgender [patients], I feel more comfortable with gay/lesbian individuals as I have had more work/social experiences with them.”

“Quite minimal. Live in a rural area. Only have had interaction with gay/lesbian patients (that I am aware of)”

### Pronouns and gender identification

Many SGM individuals, particularly those who identify as transgender, nonbinary, or genderqueer, use pronouns different from those assigned at birth, including traditional pronouns such as “he” and “she” or gender-inclusive pronouns such as “zie.” One of the most prevalent themes that emerged was providers’ concerns about using the proper pronouns for SGM patients, or clinical scenarios complicated by pronouns. Multiple providers recounted experiences of improper pronoun use in the clinical setting, as evidenced by the quote below where the patients should have had “she/her” in the medical record:

“I treated a transgender woman and all the pronouns in the notes were he/him.”

Clinicians also provided comments suggesting they had trouble keeping track of pronouns in relation to sex assigned at birth:

“We had a transgender [patient] who felt the MD was being mean by referring to **his** birth gender but it was a factor in the genetics of **her** disease.”

One provider relayed a similar experience and highlighted weaknesses in the healthcare system that contribute to the problem:

“… Also no obvious area in … patient’s EMR to identify their gender identity/preferred (sic) pronouns. I would hope this would be something that would be listed right next to something as important as their DOB.”

Some providers focused not on pronouns directly, but rather on institutional barriers related to gender identity, particularly among transgender patients:

“Screened a [transgender] patient for an oncology clinical trial, neither the physicians at our hospital, nor sponsors with the drug company, could say with conviction if we should enroll the patient according to her presenting gender identity or gender assigned at birth. Ultimately, the patient declined being screened for the study because of the hesitation regarding treatment. I believe we did the patient a disservice.”

### Perceived patient attitudes

When asked about reservations in treating SGM patients, a minority of respondents made assumptions about SGM patients’ previous negative experiences with healthcare providers:

“More suspect of health care providers”

“Due to discrimination, the LGBTQ patients I have worked with are very hostile at first expecting they are going to be treated differently and judged.”

### Positive experiences

Although many providers focused on challenges they faced with SGM patients, others recounted favorable encounters:

“I worked in an AIDS clinic for 16 years and had many wonderful experiences with the LGTBQ population. They taught me may things!”

*“Treating LGBTQ patients can be very rewarding …”*

*“Excellent experience with the LGBTQ community[.]”*

*“My experience with this patient [population has] been positive.”*

### End-of-life care

Three respondents recounted their own experiences with end-of-life care in SGM patients:

“I have a female patient with advanced lung cancer who has adult children from a former male partner. She has a female partner now that she’s been … with for 18 years. The patient has estranged relationships with some of her adult children because of this. It is important to understand the personal/social issues our patients are going through in order to provide the best care. At some point, this patient will encounter end-of-life issues, and her family dynamics will be an issue and a worry for her.”

“Treating terminal cancer patients, it was important to know about decision makers and ensure the patient has a living will.”

“Have treated LGBTQ patients with AIDS/HIV and assisted with End of Life Care. Majority of time [the patients were] alone at the End of Life.”

### Clinical care

While some providers had little to no experience with SGM patients, others saw them regularly. Such providers reported difficulty determining when to apply institutional sex-based policies among SGM patients:

“Sometimes we have a hard time convincing lesbian women about getting a pre-study urine pregnancy test. They insist they are not pregnant and haven’t had sex with a male. But I tell them [it’s] an institutional policy …”

Other respondents highlighted clinical scenarios in which sexual and/or gender orientation were objectively and inextricably linked to patient care:

“I have seen a couple of patients that wish to convert from a female chest to a male chest hoping that [bilateral] mastectomies for high risk would achieve the desired cosmetic appearance”

Other respondents described situations in which it seemed imperative to know a patient’s sex at birth:

“In radiation oncology practice, received a referral on a gender-reassigned individual for squamous cell ‘cervical’ cancer. No mention in the [medical] record that this patient was male at birth and ‘cervix’ was actually penile tissue transplanted in gender-reassignment surgery. In calculating drug dosing (e.g. carboplatin)[,] [estimated glomerular filtration rate, a measure of kidney function] is different for males/females. QTc [an interval on an electrocardiogram] ranges are different for males/females. I believe it’s important to know if the patient’s organs are male organs or female organs.”

“Was surprised by my [patient’s] gender at the time of surgery when a Foley [catheter] was being placed. This led to a potential crisis of … [misidentification].”

Still others asked questions about SGM-specific clinical needs in the oncology setting:

“I treat breast cancer patients and while I have not treated a transgender patient, I would think that lowering a patient’s estrogen levels to avoid cancer recurrence could negatively impact a transgender patient’s quality of life. I would be interested in knowing what other clinicians do in this scenario.”

“I work with survivorship and feel there should be a booklet on sexual problems that they may face. For instance: Are there issues with postmenopausal women and vaginal dryness for lesbians?”

### Education and training

While a vast array of additional themes emerged, perhaps the most unifying was the recognition that more education and training for providers on SGM healthcare is needed. When asked, “What suggestions do you have for improving the cancer care of the LGBTQ population?,” 97 of 184 responses were related to this need:

“I think that there should be mandatory training on different things we should be aware of when interacting with the LGBTQ population.”

“Education in all healthcare settings regardless if healthcare setting is backed by a religious organization”

“Training and ensuring all providers and staff are aware of appropriate interactions. We have had nurses who have worked hard to ensure all staff address transgender patients appropriately. Everyone should be responsive without a nurse having to be the champion for the transgender patient any more than they are champions for all patients.”

“Sensitivity training is a must”

“Educate providers on sensitivity to the topic. If they need specifically different care, publish in [the National Comprehensive Cancer Network] guidelines or update them.”

“As a part of the LGBT community myself, this survey is making me aware of my own lack of knowledge regarding the health disparities and challenges that the LGBT community might face, so I would be really interested in seeing healthcare providers educated on these issues.”

### Building rapport

Many providers also highlighted ways they attempt to connect and build rapport with their SGM patients. These efforts included using inclusive language, disclosing their own identities as SGM when applicable, and getting personally involved in the SGM community:

“I am gay and I would think very inclusive. I use open conversation (a/k/a do you live with a loved one?) … I recently had a gay man, after I gently coaxed that he had a partner/male, and then I shared that I had a husband …”

“I have many [LGBTQ] friends and have tried to be an active part of the community”

“I am a Gay male physician and have significant involvement in my community, medical center and medical school in relation to LGBTQ issues, education and awareness”

Few providers demonstrated negative attitudes toward this population, exemplifying ways to not build rapport:

*“I personally think it is wrong”*

*“Don’t be so sensitive, stop [having] a victim attitude”*

## Discussion

Building on our prior work conducted among oncologists at NCI-Designated Cancer Centers, the current study was conducted among oncologists, nurses, and physician assistants within the ECOG-ACRIN Cancer Research Group. As such, the goal of this study was to identify the range of oncology care providers’ experiences with, reservations toward, and suggestions for improvement in SGM cancer care to generate potential targets for intervention to improve care for this underserved population. Almost half of the 490 respondents provided at least one answer to an open-ended question, and together these responses evoked several common themes. Respondents reported largely positive or neutral experiences with SGM patients, with very few outright negative attitudes toward this population.

Many respondents described a lack of exposure to SGM patients, most notably transgender patients; with this came provider concerns about correct pronoun use among transgender patients. A lack of experience with transgender patients has been seen in our group’s previous studies; however, this concern for pronoun use is more prominent in the current study (Shetty et al., 2016; Schabath et al., 2019; Sutter et al., 2020). This may reflect the growing cultural sensitivity surrounding SGM-specific issues in society as a whole – i.e., providers were familiar enough with transgender issues that many of them independently recognized the more nuanced topic of pronouns as a challenge facing this population. This awareness of pronouns as an issue in SGM health was also seen in a recent survey of medical students, wherein most participants believed incorrect pronoun use may lead to patients’ nondisclosure of SGM status (Jamieson et al., 2020). However, these same findings demonstrate there is still room to grow in competence with respect to caring for SGM patients.

Other studies of healthcare providers and transgender patients have confirmed these shortcomings and demonstrated that they serve as barriers to care. For example, Sanchez et al. noted that the most frequently reported barrier to care among male-to-female transgender patients surveyed was access to a provider knowledgeable about transgender health issues (32%), followed by access to a transgender-friendly healthcare provider (30%; Sanchez et al., 2009). A study of transgender youths and their caregivers confirmed that inconsistent use of one’s chosen name and/or pronouns was a major barrier to care (Gridley et al., 2016). A recent survey of oncologists in the United Kingdom showed that 49% of surveyed providers never asked a patient’s gender identity, 64% never asked a patient’s pronouns, and 87% stated they always or often assumed a patient was cisgender (Berner et al., 2020). Among gay men and lesbian women, interactions with healthcare providers who demonstrated fear of behaving incorrectly hindered communication with providers (Röndahl et al., 2006); this provider fear may apply to the use of gender pronouns as well.

In addition to these highly prevalent themes of lack of experience and challenges with pronouns, smaller numbers of providers raised two unique considerations: perceived distrust of providers among SGM patients and end-of-life care. Regarding the former, providers’ perceptions of SGM patients’ hesitations is not commonly surveyed, but anecdotal reports of hostility and suspicion toward healthcare providers may be rooted in previous negative experiences with healthcare providers. A series of studies by Nadal et al. identified microaggressions that SGM people face, such as use of heterosexist terminology and endorsement of heteronormative culture, as well as common SGM responses to these microaggressions including behavioral, cognitive, and/or emotional reactions (Nadal et al., 2011a,b, 2016). Although these studies were not exclusively conducted in the healthcare setting, other studies have confirmed that SGM patients face similar microaggressions from – in addition to overt discrimination by – healthcare providers (Dean et al., 2016). Thus, we hypothesize that suspicion toward healthcare providers is a product not of sexual or gender orientation *per se*, but of previous negative experiences.

With regard to end-of-life care, respondents noted challenges related to advance directives, decision-making, and family dynamics. Although the end of life can be physically, emotionally, and ethically challenging regardless of a person’s sexual or gender orientation, SGM patients face their own unique concerns at this juncture (Sprik and Gentile, 2020). The responses here highlight some of the nuances to end-of-life care in SGM patients. For example, they may face homophobia from healthcare providers (Bristowe et al., 2016); may avoid end-of-life healthcare altogether due to previous discrimination by healthcare providers (Bristowe et al., 2018); and often encounter legal and financial barriers related to lack of relationship recognition (Bristowe et al., 2016, 2018; Sprik and Gentile, 2020). End-of-life care is a fundamental component of many cancer patients’ journeys. Therefore, to more fully care for SGM patients at the end of life, oncology providers must understand their SGM patients’ relationships with their partners and families and any system barriers, which requires patient-provider trust and rapport. In-depth goals-of-care discussions, which may or may not include concerns directly related to SGM status, must be an active component of end-of-life care. Training in culturally responsive care and cultural humility, involving components of knowledge, self-reflection, and active listening, has been proposed to reduce SGM health disparities at the end of life, though proper care at this essential juncture will require provider engagement and enthusiasm as well (Sprik and Gentile, 2020).

A larger proportion of providers mentioned aspects of clinical care specific to SGM populations that they found challenging, ranging from screening guidelines to sexual health. The findings from the current study confirm our previous findings of oncologists at NCI-Designated Cancer Centers where providers requested increased dissemination of guidelines for screening and treatment of various conditions in this population (Sutter et al., 2020). Furthermore, we previously demonstrated lack of knowledge of appropriate screening practices in SGM patients (Tamargo et al., 2017; Schabath et al., 2019).

Largely in response to such limited knowledge in treating SGM patients with cancer, the single most important theme that emerged from the qualitative responses in the current study was the need for increased provider education and training. Thus, there is a pressing need for curriculum development to address cancer disparities in SGM patients and to promote culturally responsive care. Provider training programs have been developed by the Fenway Institute and National LGBT Health Foundation, but training specifically for oncology providers has been limited. The Curriculum for Oncologists on LGBT populations to Optimize Relevance and Skills (COLORS) training program was developed for this purpose, and offers modules focused on SGM basics, inclusive environments, initiating oncology care with SGM patients, and issues in cancer survivorship among SGM patients (Seay et al., 2020). Training programs like the online Educating Nurses about Reproductive Issues in Cancer Healthcare (ENRICH) effectively engage non-physician oncology care providers as valuable team members and may improve the healthcare experience of SGM populations (Quinn et al., 2019; Sutter et al., 2020).

Some providers highlighted an additional need for institutional and policy changes to further SGM oncologic health. Multiple providers mentioned challenges in enrolling transgender patients in clinical trials, citing lack of clarity regarding whether transgender patients were eligible for studies and regarding how to classify transgender patients in terms of gender. Although to our knowledge there has not been research further delineating or quantifying these limitations to clinical trial enrollment, multiple studies have identified other institutional barriers to SGM health. One major barrier is a lack of concrete screening guidelines for SGM patients, especially transgender patients, as most published guidelines are based on cisgender patients (Haviland et al., 2020); furthermore, it may be more difficult for transgender patients to get appropriate screening tests approved if such screening tests are recommended for the opposite gender (Agénor et al., 2015). Thus, in addition to needing improved education and training for providers, institutional policy changes are needed to provide better SGM healthcare. Another institutional barrier is lack of collection of sexual orientation and gender identity data (SOGI) in the medical record (Institute of Medicine Committee on Lesbian G, Bisexual, and Transgender Health Issues and Research Gaps and Opportunities, 2011; Alexander et al., 2020); the Centers for Medicare and Medicaid Services and the Department of Human Services now require electronic health records to include structured fields for SOGI data, but barriers to thorough and consistent collection remain, and many prominent cancer registries do not include SOGI data (Burkhalter et al., 2016). Furthermore, while some institutions have non-discrimination policies, it is often unclear who can access SOGI data or that a patient has a right to verbally relay this information and not have it in their medical records (Thompson, 2016; Brooks et al., 2018).

A final theme highlighted in this study centered on providers’ efforts to build rapport with their SGM patients through both their one-on-one interactions with patients and their involvement in the SGM community. Encouragingly, these reported provider behaviors reflect greater acceptance of SGM patients – this increased acceptance is also supported by the many positive experiences respondents recounted. These themes together suggest provider desire and enthusiasm for improving one’s ability to care appropriately for SGM oncologic patients. This desire and enthusiasm may enhance the effects of knowledge and training in culturally responsive care and significantly improve the experience of SGM patients, as the success of such training depends also on the providers undertaking it.

We acknowledge several limitations to the study, most importantly the moderate response rate (46.5%) to qualitative questions among survey respondents. Additionally, although approximately 4.5% of the population identifies as LGBTQ, 8.7% of respondents stated they were lesbian, gay, or bisexual, suggesting that a disproportionate number of respondents identify as SGM (Conron and Goldberg, 2020). This may contribute to nonresponse bias, with those less familiar or less comfortable with SGM patient populations or alternatively do not believe this is a significant care delivery issue being less likely to respond. Clinicians more invested in SGM health disparities and/or healthcare delivery, including those who themselves identify as LGBTQ, may have been more likely to complete the survey, particularly the optional qualitative questions. Additionally, the large proportion of positive or neutral to negative responses may reflect social desirability bias, in which survey respondents answered questions in ways more likely to be viewed favorably (Hebert et al., 1997).

Overall, the qualitative comments of this survey highlight oncology care providers’ need for increased exposure to and training on SGM cancer care and culturally responsive care. This and our prior studies demonstrate that oncology care providers are not only willing to engage in such training, but also independently recognize this need. Furthermore, this training should extend beyond physicians and include the broader healthcare team to influence the most meaningful change.

## Data availability statement

The raw data supporting the conclusions of this article will be made available by the authors, without undue reservation.

## Author contributions

CLT, MBS, and GPQ were responsible for conceptualizing the article, performing the formal analysis and all methodology, project administration, resources, software, supervision, validation, and visualization. EPM, LW, MAS, RCC, BJG, and MBS curated the data and acquired funding. All authors contributed to the article and approved the submitted version.

## Funding

This study was conducted by the ECOG-ACRIN Cancer Research Group (Peter J. O’Dwyer and Mitchell D. Schnall, Group Co-Chairs) and supported by the National Cancer Institute of the National Institutes of Health under the following award numbers: UG1CA189828, UG1CA233320, UG1CA233160, UG1CA233180, and UG1CA233341.

## Conflict of interest

The authors declare that the research was conducted in the absence of any commercial or financial relationships that could be construed as a potential conflict of interest.

## Publisher’s note

All claims expressed in this article are solely those of the authors and do not necessarily represent those of their affiliated organizations, or those of the publisher, the editors and the reviewers. Any product that may be evaluated in this article, or claim that may be made by its manufacturer, is not guaranteed or endorsed by the publisher.
