# Supplementary material for: “I need more knowledge”: Qualitative analysis of oncology providers’ experiences with sexual and gender minority patients
Source: Front Psychol. 2022 Aug 15;13:763348. doi: 10.3389/fpsyg.2022.763348 (PMC9421156; doi:10.3389/fpsyg.2022.763348)
Supplement: Supplementary file 1 [file Table_1.DOCX]

| Supplementary Table 1. Thematic matrix of themes and responses | | |  |
| --- | --- | --- | --- |
| Emergent Themes | **Question**^a^ | **Responses** | |
| Lack of experience | 1 | I have no experience speaking with patients of the LGBTQ community. If I did, it was not my knowledge  Don't have any  Have only had a few pts.  I have had limited experience with transgender, I feel more comfortable with gay / lesbian individuals as I have had more work / social experiences with them  I am a cancer doctor - so I do not deal with some of the issues you describe regarding screening (i.e. I do not see them until they get cancer).  I don't currently treat patients.  I don't have experience  I have little experience.  I personally have not had any experience as my position at this facility is primarily data collection and regulatory.  No experience with LGBTQ patients  none that I can think of that are important  None that I know of  none to report  Nothing stands out  Quite minimal. Live in a rural area. Only have had interaction with gay/lesbian patients (that I am aware of)  Thx for working on this. Perhaps include working with transgender colleagues as well. /  transgender patients are not as common in our clinic, so it is something that people often don't know how to handle  I'm a clinical research assistant so I do not have much experience  N/A (8 respondents)  None (16 respondents) | |
|  | 2 | haven't really been confronted with it | |
|  | 3 | Not sure at this time, I know what has been within the media, just not necessarily my experience.  I really don't know what the issues are with the cancer care of the LGBTQ population so I don't have suggestions.  I'm unaware of the current issues | |
| Pronouns and gender identification | 1 | need to know birth gender as well as current gender identification, and how to find this out from patients /  Most experiences have gone well. We believe in treating all patients with dignity and respect. Patients are grateful when we ensure they are addressed by pronouns reflecting the gender identity they identify with.  I treated a transgender woman and all the pronouns in the notes were he/him.  I have noticed a constant use of incorrect pronouns from all aspects of the medical team, including myself, when addressing a trans patient of mine. Also no obvious area in said patient's EMR to identify their gender identity/preferred pronouns. I would hope this would be something that would be listed right next to something as important as their DOB.  Gender pronouns can be difficult especially when treating a patient for a diagnosis consistent with the gender of birth when they present as the other sex.  We had a transgender pt who felt the MD was being mean by referring to his birth gender but it was a factor in the genetics of her disease.  Screened at MTF patient for an oncology clinical trial, neither the physicians at our hospital, nor sponsors with the drug company, could say with conviction if we should enroll the patient according to her presenting gender identity or gender assigned at birth. Ultimately, the patient declined being screened for the study because of the hesitation regarding treatment. I believe we did the patient a disservice.  I've provided direct care to transsexual *(sic)* patients for decades. They have been more easily identifiable as such as opposed to other gender identities.  This is an experience from when I was working checking patients in for all outpatient testing. A transgendered *(sic*) person complained that they were questioned about their identity because the person's appearance was not matching the appearance and sex on the patient's ID. My coworkers and I felt bad about this because there were relatives checking in patients all the time, so it wasn't usual for us to question who the patient is. How should we have approached this patient?  In past experiences I found that the patient no matter what sex they were born prefers to be acknowledged with the sex they determine to be. They usually do not like questions regarding their sexual orientation.  Was surprised by my patients gender at the time of surgery when a foley was being placed. This led to a crisis of potential patient misidentification  When screening patients for potential clinical trials and it's noted in the chart that patient is transitioning... What would be the appropriate term to use, what communication would be helpful or relevant to better understand the patient.  A subject enrolled in a female only breast cancer biomarker study as she was registered in our EMR as a female. It wasn't until after enrollment and study procedures that we found out in fact she was transgender - the Sponsor required us to withdraw the subject.  I’ve also noticed that many providers may need more training on sensitivity to gender identity; I've witnessed instances where a provider does not respect/honor their patient's wishes to identify with their pronoun of choice due to "medical reasons". An example would be when they refer to their male-assigned-at-birth patient as "him" when they identify as "her", even when in their presence. Unless the topic is gender relevant (i.e. prostate or ovarian disease) then people should respect the gender the patient identifies with and call them by their preferred pronoun etc.  I generally ask patients in a non-judgmental way to describe their gender identity. I find most are willing to tell me how they see themselves and what sexual partners and practices they have provided it is relevant to their medical care.  Let them share the relationship with their partner or the person accompanying them to their appointments. Ask how they would like to be addressed as do not always go by the name on the medical record. Treat respectfully and free of judgement as you would any patient.  In radiation oncology practice, received a referral on a gender-reassigned individual for squamous cell "cervical" cancer. No mention in the med record that this patient was male at birth and "cervix" was actually penile tissue transplanted in gender reassignment surgery. In calculating drug dosing (e.g. carboplatin) EGFR is different for males/females. QTc ranges are different for males/females. I believe it's important to know if the patient's organs are male organs or female organs. | |
|  | 2 | I am uncomfortable when a pt identifies as the opposite gender. It is hard for me to say Mr/Miss or he/she when the chart states one gender and the pt identifies as the opposite. It is also hard once the pt has started the gender changing process to know what to screen for.  Afraid I will get sued if call them by the wrong pronoun or name. | |
|  | 4 | So patient is transitioning to a female gender, but is still a male, but in process and soon to have an operation next week... On the from he/she wishes to identify in the feminine with a female name etc...on the enrollment form what would be the patients gender? | |
| Perceived patient attitudes | 1 | Some appear to expect one to be less congenial than with others.  One person was openly hostile toward a provided due to the impression that he would not approve of the person's lifestyle and preferences.  My patient with breast cancer was very angry in general. Consequently, her oncologist and I were targets of her unhappiness. She decided to withdraw from the research trial early, despite extra attention to listen and provide supportive care. Her partner returned to us for education and was very receptive.  Patient's fear of health care and judgements based on prior health care encounters. Patient's concern over maintaining employment, health insurance and housing. Concern over costs associated with transition-related expenses. Knowledge deficit on short-term and long-term care plans.  There seems to be an initial lack of trust of provider, this improves as the relationship builds | |
|  | 2 | Potential for preconceived thoughts on patients part that all providers will automatically be negative toward them. Because of the emotional charged nature of subject, potential for misunderstandings based on question asked by provider possibly coming across as ignorant or insulting.  I have very happy and open to treating LGBTQ patients, however I am thin, white, hetero cis male and I feel that many patients will avoid or be hesitant to be open to me based on preconceptions about how other non-inclusive white males might be  more suspect of health care providers  Due to discrimination, the LGBTQ patients I have worked with are very hostile at first expecting they are going to be treated differently and judged. I have to take more time and be very careful in words and action while treating them until we can establish trust. | |
| Positive experiences | 1 | Excellent experience with the LGBTQ community  I worked in an AIDS clinic for 16 years and had many wonderful experiences with the LGBTQ population. They taught me many things!  Currently have LGBTQ patient on a clinical trial. Unaware of LGBTQ status until patient disclosed about 6 weeks after our initial visit. My experience with this patient have been positive and I am very supportive of his/her lifestyle. | |
|  | 4 | Treating LGBTQ patients can be very rewarding, and then are usually well-informed and interactive patients who engage in their treatment programs actively. | |
| End-of-life care | 1 | Treating terminal cancer patients, it was important to know about decision makers and ensure the patient has a living will.  Have treated LGBTQ Patients with AIDS/HIV and assisted with End of Life Care. Majority of time were non-compliant with treatment, anger issues and was alone at the End of Life.  I have a female patient with advanced lung cancer who has adult children from a former male partner. She has a female partner now that she's been in a partnership with for 18 years. The patient has estranged relationships with some of her adult children because of this. It is important to understand the personal/social issues our patients are going through in order to provide the best care. At some point, this patient will encounter end-of-life issues, and her family dynamics will be an issue and a worry for her. | |
| Clinical care | 1 | I was very lucky in my surgical fellowship to work with a breast surgeon and reconstructive breast surgeon who developed expertise in risk reducing prophylactic mastectomy techniques for transgendered patients which exposed me to unique oncoplastic considerations.  I have seen a couple of patients that wish to convert from a female chest to a male chest hoping that BL mastectomies for high risk would achieve the desired cosmetic appearance  It is annoying when protocols require me to test for pregnancy in my lesbian patients.  Sometimes we have a hard time convincing Lesbian women about getting a pre-study urine pregnancy test. They insist they are not pregnant and haven't had sex with a male. But I tell them it’s an institutional policy....  I treat breast cancer patients and while I have not treated a transgender patient, I would think that lowering a patient's estrogen levels to avoid cancer recurrence could negatively impact a transgender patient's quality of life. I would be interested in knowing what other clinicians do in this scenario.  In radiation oncology practice, received a referral on a gender-reassigned individual for squamous cell "cervical" cancer. No mention in the med record that this patient was male at birth and "cervix" was actually penile tissue transplanted in gender reassignment surgery. In calculating drug dosing (e.g. carboplatin) EGFR is different for males/females. QTc ranges are different for males/females. I believe it's important to know if the patient's organs are male organs or female organs.  I work as a general and pediatric Urologist in an academic institution that treats a very high number of patients with Disorders/Differences of Sex Differentiation, forming part of a multi-disciplinary based care environment. These cases are only treated as part of an MDT-based approach.  I work with survivorship and feel there should be a booklet on sexual problems that they may face. For instance: Are there issues with postmenopausal women and vaginal dryness for lesbians?  We live in an area that has a higher demographic of gay males. I feel it makes a big difference when a provider considers this and their sexual activity and lifestyle so they can take extra care to screen for HPV related disorders and malignancies (like anal cancer), and other diseases that may be associated/prevalent in the population such as polycythemia secondary to steroid/hormone use.  - We had a patient who was born a male but changed to a female, she had free silicone injections on her breast and another body part and then developed breast cancer. She did not want to have surgery, the surgeons were nervous about surgery due to the free silicone. Her treatment was challenging. | |
|  | 2 | Shared decision-making paradigms regarding breast surgical choice and reconstructive options should include exploration of gender-based preference.  I would be worried with transgender patients and if they're on hormone therapy/treatment and if that may impact any prescribed drugs. I also wonder about hormones and if they can increase or decrease risk for certain cancers. For example a [M-to-F] transgender person who's on hormone therapy. How often would they need to be screen for prostate cancer? And when should they be screened for breast cancer, as it can affect both men and women.  only as it may relate to ability to manage medical problems in an individual pt.  I have absolutely none. I am a physician - I am here to help people regardless - I took that oath 40 years ago and I live by it. / With regard to those in the LGBTQ group without identifiable endocrine disorders, I DO have discomfort about the regular use of surgery to remove organs or make organs to try to match, as you say, "their outsides to their inner sense of self". This is something that requires extensive psychological input. In fact, these people are termed to have "gender dysphoria". I have difficulty as a doctor seeing those people have surgical removal of their sex organs etc.  Our institution is involved in Male to Female transitioning and as such sometimes treat trans-gender female patients for prostate disease, both benign and malignant. This can be challenging in terms of interaction with staff that may have bias or other patients that may be biased towards these patients. | |
|  | 3 | Having a FTM transgender child I try to instill the fact they still need female organ cancer screenings, this is something that is a challenge for them to acknowledge so providers must be sensitive on how to approach and speak with them not just to schedule but during the procedures. | |
| Education and training | 1 | All staff should undergo mandatory training in providing care to our LGBTQ community. We should be a safe haven for this population.  I am totally accepting of the LGBT population but admit I have a lot knowledge gaps.  I had an AYA gay man being treated for cancer. He needed to be taught about safe sex and avoiding infection. He was in a monogamous relationship at the time. No one on our team other than myself was comfortable enough to speak with him regarding this topic. I was able to provide some education and precautions to avoid infection but in questioning other colleagues about teaching, no one had information about guidance for chemotherapy exposure in semen (or vaginal fluid) for a gay couple. There is a lot of information regarding not getting pregnant or impregnating a woman while receiving chemotherapy, but not for risk of chemo exposure in general during sexual activity. I would have liked to have more information to feel more competent and informative in my teaching.  appreciated a session at one of the ECOG meetings that talked about LGBTQ issues and pt population. URCC also has an interest in this topic  Patient's fear of health care and judgements based on prior health care encounters. Patient's concern over maintaining employment, health insurance and housing. Concern over costs associated with transition-related expenses. Knowledge deficit on short-term and long-term care plans. | |
|  | 2 | need to know more to be culturally sensitive  have none, just would like more education how to best help communication with patient  I have been exposed to and familiar with the LGB population my whole life, however I do need more education/experience on the needs of the TQ population. My only reservation would be my ignorance on the topic, I would not want to offend anyone unintentionally or overlook their needs.  No reservations...I feel I need more training on healthcare issues and how to discuss healthcare needs  Simple lack of knowledge for LGBTAIQ specific needs. This is however something that I would hope can be addressed through education.  I don't have enough knowledge of things that I should watch for  none other than lack of knowledge  no sure the associate diseases and risks for specific populations  My knowledge deficit is an obstacle. / I also feel I live in a very conservative area where LGBTQ may not feel as safe being open as other places- but I guess this is not a reservation. /  Not being educated about the LGBTQ community, especially transgender.  My only reservation is that my cultural competence is not as good as it should be, but mostly I am comfortable. Regarding trans patients, I have no experience.  Not having enough knowledge to adequately treat and respect their culture.  I have no reservations in treating this population. However, just like we have diversity training to properly care for those of different cultural backgrounds, it would be beneficial to all staff to have training on the needs of the LGBTQ population.  Just better understanding  I feel the LGBTQ community has a barrier and are afraid to be open with health care providers in fear of being judged or their concerns not being taken serious enough. I also believe the lack of knowledge for those transitioning and are on hormone therapy. And how to help the patient during the change of their physical and emotional journey. Many of us after had that experience to relate to.  Only reservation is need for more education and feeling unsure of myself.  Would like more updated information on medical care and needs of the specific populations so best care can be provided.  My only reservation is lack of knowledge around specific needs.  If there are special health needs, these were not addressed in nursing school, so I am not well educated in special LGBTQ health needs.  no reservations but would like more information to better serve this population.  worry I do not have all info  I simply need more knowledge, particularly related to cancer care in transgender patients.  Would like better education as to what their needs are, fears, expectations. What limitations or barriers do they see to seeking or getting health care & cancer care? Body image considerations dealing with different diseases, especially cancer, breast cancer, prostate, testicular cancer etc.  I would welcome more education and instruction on discussing relevant issues  Reservation would be lack of knowledge surrounding this topic.  lack of knowledge  none, just would appreciate any education to that I can fill any knowledge gaps I have  lack of my knowledge regarding specific health problems  None, other than it being sometimes awkward to as I am not well versed in the LGBTQ community, but would like to be respectful and inclusive  unfamiliarity with health issues of this population.  My personal lack of knowledge could be seen as offensive. | |
|  | 3 | I believe that centers should consider informational sessions on LGBTQ health and then gain some level of certification that can be easily found by patients seeking care.  I think that there should be mandatory training on different things we should be aware of when interacting with the LGBTQ population.  staff/provider education  more provider education  Education and nonjudgmental attitudes required.  Try to provide educational sessions via webinar, etc. partner with groups  Keep educated  education of everyone involved  more education for faculty and staff  More transgender care education  education for caregivers  continue education of cancer risk factors  Education in all healthcare settings regardless if healthcare setting is backed by a religious organization  Teaching modules that incorporate these topics.  awareness and workplace training  Education of staff regarding common practices among this community, rather than what they read or hear.  Have much more education around this topic. Understanding and having greater knowledge helps in making sure that all LGBTQ persons get the best care.  Education, trials, support.  Training and ensuring all providers and staff are aware of appropriate interactions. We have had nurses who have worked hard to ensure all staff address transgender patients appropriately. Everyone should be responsive without a nurse having to be the champion for the transgender patient any more than they are champions for all patients.  Educate providers on sensitivity to the topic. If they need specifically different care, publish in NCCN guidelines or update them.  Sensitivity training is a must  More education  Provide more education to caregivers  Education of the staff  Provide education to providers  Increased education on things that I should know.  any education  I feel educational needs for our LGBTQ population is the important part of helping this population  Improving education of staff - providing save places for the LGBTQ population to receive care. This would foster a better relationship to ensure follow up care  I try to approach all my patients as human beings. There are so many axes of difference and need - I am open to learning about how sexual and gender identity influence these needs, but if honestly doesn't change dramatically how I approach cancer management. Good communication/openness and trust are key.  education / cultural competency  Not sure / more main stream education.  More inclusive practices to encourage education about need for screening and surveillance  education of physicians and HealthCare workers  improve cultural competence of the entire healthcare machine  Becoming more familiar with this patient population and their health care needs.  Providing more education and making it a part of hospital orientations, degrees, etc.  more education on LGBTQ issues for all staff that interact with patients  education to providers about LGBTQ special considerations for care of these patients.  More education on how to discuss gender identity and sexual orientation with patients.  Self-teach computer information modules would be helpful  Staff education. Guest speakers of patients from the LGBTQ community  As mentioned before there is a need for more education.  more training as always  Training to health care provider and educate the community  Development of guidelines for additional screening/treatment considerations in LGBTQ patients  Have much more education around this topic. Understanding and having greater knowledge helps in making sure that all LGBTQ persons get the best care.  Education/ diversity training for health-care professionals.  improved education of health care providers  Education around how to approach questions and surveys to help understand risk factors for disease  Education of healthcare staff.  Offer courses on special needs for LGBTQ  Education. Hearing what that population needs in their own terms.  National SOGI data is a must, in order to learn more about the cancer experiences of this community. We really need to improve trans people cancer screening since many retain organs that need to be screened, we really don't know the long term biologic effects of hormones for decades on these organs in trans people, trans people have higher rates of alcohol, tobacco, and illicit drug use, which can increase cancer incidence. And training every touch-point in health care for LGBTQ pts is a must (schedulers, nursing and MD staff, etc.) to ensure culturally competent, compassionate, and state of the art cancer care.  I think educating the care givers about the health needs/concerns of the LGBTQ+ patients, and what their biggest fears are when facing cancer care and health care in general. Maybe what is needed is a peer to peer support system link for patients. Improving the care of the LGBTQ+ community is something that needs to change from the tops of organizations downwards. Maybe a plan for how to implement appropriate form questions in a health care environment would also help. In my opinion you can't teach people to be more compassionate or caring. There is a class called See Me As A Person, that some institutions use, but it is not specific to LGBTQ+ patients. Maybe it would be a place to start.  Education for staff and caregivers  consistent availability of reliable information and updates  Education about preventable measures of sexually transmitted diseases or viral infections that can increase cancer incidence, safer sexual practices and awareness about screening and early detection of different cancers. Open discussion about these topics or any questions an individual can have in the general care at our clinics  Education is always good. And of course more governmental support for equity disparity NCI subcommittees would be great!  more training, especially pertaining to research patients  Maybe education on different risk factors based on LGBTQ status vs the rest of population.  more education  REQUIRED TEACHING IN MEDICAL SCHOOL AND RESIDENCIES. (I give a lecture on mammography and caring for the transgender patient every other year.)  Keep communicating and offering education.  More education so providers have more knowledge and comfort in providing care to this population with specific care needs separate from heterosexuals. More guidance on what is important to the LGBTQ community for their providers to know/ask and how to ask with confidence and without being inadvertently offensive.  Education and an atmosphere of tolerance for differences.  making education for providers easily accessible  continued education on special needs / concerns in their care that may be different from routine standards  need provider education regarding health related difficulties  increased awareness, research, and education  education on how to talk with patients across the board in meeting them for the first time and inquiring about their sexuality  Information  More awareness for transgender people so that they have appropriate care based on the gender that they are born with.  Additional education  Education especially around social issues  Screening recommendations and education on impact of hormonal treatments during gender changes in transsexuals on cancer / Risk and on the corollary-the psychological and physical effects of cancer directed hormonal therapy needs to be better understood  improved education and research studies  more education  More info on social needs  Guidelines and perhaps general Q & A. possibly scenarios as well -  Required TEACHING IN MEDICAL SCHOOL AND RESIDENCIES. (I give a lecture on mammography and caring for the transgender patient every other year.)  I know I need more knowledge.  More education on how it can be improved/ awareness  Seems like I need more information specific to this population- their needs.  I believe training would be very helpful for providers.  As a part of the LGBT community myself, this survey is making me aware of my own lack of knowledge regarding the health disparities and challenges that the LGBT community might face, so I would be really interested in seeing healthcare providers educated on these issues.  1)education of providers in ways to sensitively care for this population. I don't think this should be mandatory because I don't think people with negative attitudes forced to take some training will likely change their minds and may in fact become more negative  Providing mandatory education for all medical staff on the medical needs of LGBTQ patients, working with LGBTQ community organizations to help break down systemic barriers to care.  Develop ways to help this population feel welcome & not fearful of seeking health care because of fear of judgement. Include in center promotional materials. Support services. The use of pronouns and what name they would like to be addressed as during intake. Would like better education as to what their needs are, fears, expectations.  Education, communication, awareness and consideration  training is important but also visibility and interactions with LGBTQ people  Education (6 respondents) | |
| Building rapport | 1 | Exhibit acceptance and openness  I have many LGBTQ friends and have tried to be an active part of the community  Respect, tolerance and understanding of everybody needs are important to be considered including the sexual orientation as part of a good health care program.  I am a Gay male physician and have significant involvement in my community, medical center and medical school in relation to LGBTQ issues, education and awareness  I am gay and I would think very inclusive. I use open conversation (aka. do you live with a loved one?), etc. | |
|  | 3 | training is important but also visibility and interactions with LGBTQ people  Openness in both professional engagement including literature available for support groups accompanied with mission  Funding for LGBTQ-specific health care entities, as care is often not sought due to perceived or experienced discrimination by health care professionals.  Partnering with the local community, more research on the cancer related healthcare needs of the LGBTQ population, better outreach/visibility of the cancer center, clear nondiscrimination policies visible and posted throughout the clinic/hospital  market services at LGBTQ events, Pride events, places catering to LGBTQ persons  outreach to this population to both hear their concerns and establish more trust of the medical establishment  Providing mandatory education for all medical staff on the medical needs of LGBTQ patients, working with LGBTQ community organizations to help break down systemic barriers to care.  Develop ways to help this population feel welcome & not fearful of seeking health care because of fear of judgement. Include in center promotional materials. Support services. The use of pronouns and what name they would like to be addressed as during intake. Would like better education as to what their needs are, fears, expectations. . The Midwest (where I am located) does not prioritize this population. | |

^a^1: “Please describe any personal experiences treating LGBTQ patients that you consider important or informative”; 2: “Please explain any reservations in treating the LGBTQ population”; 3: “What suggestions do you have for improving the cancer care of the LGBTQ population?”; 4: “Please provide any additional comments.”
